# Potential use of 3D-printed graphene oxide scaffold for construction of the cartilage layer

**DOI:** 10.1186/s12951-020-00655-w

**Published:** 2020-07-14

**Authors:** Zhong Cheng, Li Xigong, Diao Weiyi, Hu Jingen, Wang Shuo, Lin Xiangjin, Wu Junsong

**Affiliations:** 1 Department of Orthopedic, First Affiliated Hospital, College of Medicine, Zhejiang University, Qingchun Road 79, Hangzhou, 310003 People’s Republic of China; 2 The Sport Medicine Center of the First Affiliated Hospital, College of Medicine, Zhejiang University, Qingchun Road 79, Hangzhou, 310003 People’s Republic of China

**Keywords:** 3D printing, Graphene oxide, Cartilage, Chondrocytes

## Abstract

**Background:**

Three-dimensional (3D) printing involves the layering of seed cells, biologically compatible scaffolds, and biological activity factors to precisely recapitulate a biological tissue. Graphene oxide (GO), a type of micro material, has been utilized as a small molecule-transport vehicle. With the proliferation of GO, the biocompatibility of chondrocytes in a microenvironment constructed by 3D printed scaffolds and GO is innovative. Accordingly, we speculate that, as a type of micro material, GO can be used with 3D scaffolds for a uniform distribution in the cartilage layer.

**Results:**

A qualitative analysis of the chondrocyte-proliferation potential revealed that the culture of 3D printing with a 10% GO scaffold was higher than that of the other groups. Meanwhile, the progress of cell apoptosis was activated. Through scanning electron microscopy, immunofluorescence, and in vivo research, we observed that the newborn cartilage matrix extended along the border of the cartilage and scaffold and matured. After an analysis with immunohistochemical staining with aggrecan and collagen I, the cartilage following the 3D-printed scaffold was thinner than that of the 3D-printed GO scaffold. Furthermore, the collagen I of the cartilage expression in treatment with the GO scaffold was significant from week 2 to 6.

**Conclusions:**

The findings indicate that a 3D-printed GO scaffold can potentially be utilized for the construction of a cartilage matrix. However, the optimum concentration of GO requires further research and discussion.

## Background

Graphene oxide (GO) has been utilized as a small molecule-transport vehicle as it has efficient loading and is hepatotoxic [1]. Graphene comprises carbon atoms with a honeycomb-like two-dimensional structure. Essential GO characteristics include sp^2^ carbon domains and a large surface area. The distinctive π–π stacking interaction makes GO efficient, with a large specific surface area for a high loading and absorption capacity [2, 3]. The interaction between its electrostatic and hydrophobic π domains can activate its negatively charged domains and allow efficient protein absorption to the GO via the inner hydrophobic regions [2, 4].

However, GO characteristics have not been fully elucidated because information regarding its cell responses at the early stages of tissue development remains insufficient. The biocompatibility of GO and its derivatives should be investigated through in vitro cell cultures and in vivo animal models [5].

Three-dimensional (3D) printing has become particularly important in recent years [6]. It has already been incorporated into surgical planning, education, and implant customization [7], with particular potential for tissue-engineering utilizations. Hence, it enables researchers to create precise layers of seed cells, biologically compatible scaffolds, and biological activity factors to recapitulate a biological tissue. Furthermore, the technology for depositing extracellular matrices, biochemical factors, living cells, biomaterials, or drugs onto a substrate has already been developed [8, 9]. Consequently, such a technological innovation has led to the creation of many groundbreaking treatments and equipment.

For instance, current researchers have used GO technology to biofabricate autonomously shaped and formed tissue constructs with structural integrity. This can be attributed to the recent advancements in 3D bioprinting technology, e.g., powder-bed fusion, vat polymerization, binder jetting, and material extrusion, which are distinct additive-manufacturing technologies [10].

The abilities to print biocompatible, tissue-specific matrix scaffolds with GO, and to incorporate seed cells, biological proteins, and 3D-printed scaffolds have been applied in a wide variety of fields, particularly in tissue engineering and orthopedic applications [11, 12]. An articular cartilage matrix has a low intrinsic repair capacity due to its lack of blood vessels as well as its sparse cell population. To date, the approaches for articular cartilage-matrix regeneration mainly include drug-delivery treatments, allogeneic cell-based therapies, autologous chondrocyte implantation, and osteoarticular autografts [13, 14]. Critical molecule signals can activate the molecular and cellular processes for both cartilage-matrix regeneration and degradation [15].

These treatment processes may also be associated with additional surgical risks and additional time to regain joint function, and often do not offer a long-term clinical solution. Moreover, several disadvantages of the combination of cells and biomaterials have been identified, e.g., weak mechanical strength and stability. GO is also difficult to handle and form into cartilage-matrix regenerative constructs with the desired internal structure and external shape [16, 17]. Accordingly, we speculate that as a type of micromaterial, GO should be combined with a 3D scaffold for uniform distribution in the cartilage layer.

In this study, the basic-material scaffolds, with printed collagen-chitosan as the control group, were compared with a 3D-printed GO scaffold regarding the following key research points:

1. Whether a 3D-printed GO scaffold can attach chondrocytes;
2. The decomposition progress of GO in a 3D-printed scaffold;
3. The way newborn cartilage tissues creep onto the GO scaffold.

Meanwhile, cartilage regeneration was observed, and the procedure of the newborn cartilage-tissue model is summarized herein.

## Materials and methods

### Graphene oxide (GO) characterization

GO samples were purchased from Chengdu Organic Chemicals Co., Ltd. (Chengdu, China). The particle-size distribution of the GO was recorded with a zeta electric potential-based spectrophotometer (Zetasizer 3000 HSA, Malvern, UK). The morphology of a GO particle was determined using scanning electron microscopy (SEM) after coating with platinum (JSM-6701F, JEOL, Tokyo, Japan).

### GO adsorption procedure

First, 1,1-dioctadecyl-3,3,3,3-tetram-ethylindocarbocyanine perchlorate (DiI; Sigma, St. Louis, MO, USA) was used to label the GO. The DiI solution (0.3 μL × 1 mmol/L DiI solution in anhydrous alcohol) was mixed with the GO (ratio of GO to DiI was 1:1 by weight) and incubated for 12 h at room temperature. Fluorescein isothiocyanate (FITC; Thermo Scientific, Rockford, IL, USA) is soluble in anhydrous dimethyl sulfoxide (DMSO) at 5 mg/mL. FITC was diluted in a basic buffer for the coupling procedures immediately prior to use. For each 1 mL of protein solution, 50 mL of FITC solution was added very slowly in 5-mL aliquots while gently and continuously stirring the protein solution. The reaction was incubated in the dark for 8 h at 4 °C. NH_4_Cl was added to the final concentration of 50 mM. Then, the unbound FITC was separated from the conjugate by gel filtration using a fine-sized gel matrix with an exclusion limit of 20,000–50,000. The ratio of fluorescein to protein of the product can be estimated by measuring the absorbance at 495 nm and 280 nm. Then, the FITC-conjugated bone morphogenetic protein 2 (BMP2; Huaan Co., Hangzhou, China) and DiI-labeled GO were mixed and incubated for 4 h at room temperature.

The ratio of GO to BMP2 was 1:1 by weight. Using a laser-scanning confocal microscope, the BMP2 adsorbed onto the GO (FITC-conjugated BMP2) was visualized (IX81-FV1000 inverted microscope; Olympus).

### Cell co-cultures with GO

The Institute of Biochemistry and Cell Biology, Chinese Academy of Sciences, offered a chondrocyte cell line. The chondrocytes were separated when they reached 80% confluency. The culture medium was changed every 2–3 days. Using this method, the chondrocytes propagated for three generations. The chondrocytes were cultured with GO in a 37 °C and 5% CO_2_ environment. The proliferation and protein-expression parts of the cell experiments were completed in three generations of cell cultures. To evaluate the biocompatibility of the GO with the chondrocyte proliferation, immunofluorescent, toluidine blue, and safranin staining were used to display the cells within the GO scaffold.

### Bio-ink preparation

A 1% collagen type-I solution (BD Biosciences) and a 1.5% chitosan (w/v) HCl solution (0.2 mol/L), buffered at a pH of 7.2, with a 0.5-mol/L morpholinoethanesulfonic acid solution, and NaOH (1 mol/L) were mixed in a 10:1 ratio (w/w). 1-ethyl-3-(3-dimethylaminopropyl) carbodiimide and N-hydroxysuccinimide, both at 10% (w/v) in a morpholinoethanesulfonic acid solution, were mixed with the collagen-chitosan and GO solutions (1%, 3%, 5%, 7%, and 10%).

### 3D printing and culture

Regenovo Biotechnology Co., Ltd. (Hangzhou, China) helped with the 3D printing. Microscale injection systems, including a 210-μm internal diameter nozzle, were attached to reservoirs for the inks for the polylactic acid GO scaffolds. The bio-ink reservoirs and microscale injection systems were mounted with 3 axes (*x*, *y*, and *z*), and the molding speed was set to 170 mm/s.

### Scanning electron microscopy

SEM was used to examine the 3D-printed tissue (Olympus, IX83, Japan). First, all samples were freeze-dried and coated with 10 nm of platinum/palladium (Pt/Pd) by vacuum evaporation. After conductive coating, samples were carefully fixed in the copper plate one by one. The operation of the tissue was imaged at a voltage of 5kv, working current of 10 mA, working distance of 8 mm.

### Fourier-transformed infrared spectroscopy

A Nicolet 5700 spectrometer performed Fourier transform infrared spectroscopy (FTIR) on 10-mm-diameter pellets with an instrument from Thermo Electron Scientific Instruments Corp. Two milligrams of samples were mixed with 100 mg of KBr to produce the pellets for analysis. The spectra were analyzed using the EZ OMNIC software after a baseline correction was performed (Nicolet Thermo Electron Scientific Instruments LLC, Madison, WI, USA).

### Immunofluorescence

A 4% formaldehyde solution was used to fix the tissues in the printed scaffold for 10 min and then incubated in a 10% goat serum. A 1% serum albumin of bovine and 0.3-M phosphate-buffered saline glycine were used for 1 h to stop the protein interactions. The antibody was used at a dilution of 1/250 for 1 h. DAPI was used to stain the cell nuclei (blue). Then, the chondrocytes were incubated together with F-actin, aggrecan, and collagen I antibodies at 5 μg/mL at 4 °C overnight. All the antibodies were adopted from Abcam, Cambridge, UK.

### In vivo experiments

The experiments performed on rats were approved by the Zhejiang University Ethics Committee. Six 4-week-old Sprague–Dawley rats underwent 3D-printed tissue-transplantation surgery on both knees (Fig. 7a). In each rat, the 3D-printed tissue was transplanted onto the center of the femur cartilage in the right and left knees following the cartilage hole-drilling model (Fig. 5a). As a control group, six 4-week-old Sprague–Dawley rats underwent cartilage hole-drilling surgery, only on their knees.

Two rats were sacrificed, 2, 4, and 6 weeks after the experiment. The health status of the rats remained good during the cartilage regeneration after the transplanted 3D-printed micro GO scaffold. No infections or bone fractures occurred and no rats died. All femur samples were dissected before the fixation and embedding in paraffin, and stained by immunostaining. Polarized optical microscopy was adopted to compared the difference of histologic features of chondrocytes with 3D-bioprinted scaffold transplantations. Toluidine blue staining of scaffold with femur cartilage samples was adopted to compared the difference of new generated cartilage tissues.

## Results and discussion

### Morphology and characterization of GO

**Fig. 1:**
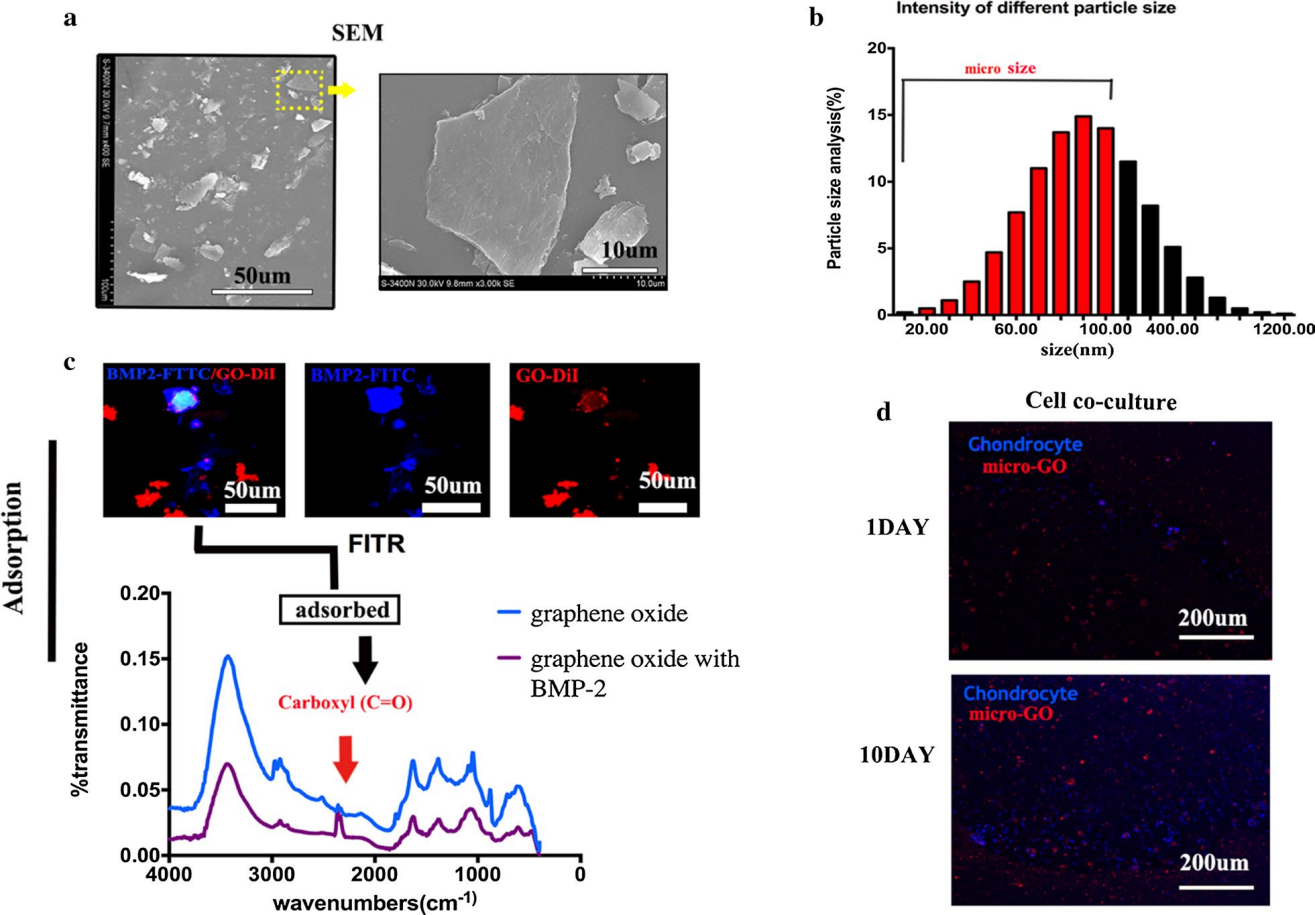
**a** Scanning electron microscopy (SEM) image showing the flake-like morphology of the graphene oxide (GO). **b** Size distribution of the GO flakes measured by an electric potential-based spectrophotometer. The size distribution showed the majority of GO was smaller than 100 μm. **c** FTIR adsorption spectra of chemical ingredients involved in the GO and bone morphogenetic protein 2 (BPM2)-GO: A peak value of 1800/cm confirms that carboxyl (C=O) played a key role in the BPM2-GO. **d** Coexistence of chondrocytes with GO: The quantity of cells around the GO increased in 10 day in the cell co-culture group

The SEM results showed that the GO had a flaky morphology (Fig. 1a). The length of the flakes in SEM was less than 100 μm. An electric potential-based spectrophotometer was used to measure the size distribution of the GO flakes. The size distribution showed the majority of GO was smaller than 100 μm. So we define the micro-GO as the size smaller than 100 μm (Fig. 1b). Subsequently, the FITC-conjugated BMP2 (green) and the DiI-labeled GO (red) were incubated.

Thus far, a variety of micromaterials that can be considered as matrices have been manufactured [18]. GO has received enormous attention in the bone-tissue engineering field owing to its unique sp^2^ carbon domains and large surface-area structure. Laboratory and clinical breakthroughs have not yet been made, although GO has been transplanted in mammals.

### Exogenous bone morphogenetic protein 2 adsorbed onto GO

An analysis revealed that the BMP2 adsorbed onto the GO, suggesting that the GO was efficiently bound to the BMP2 protein (Fig. 1c). The chemical ingredients involved in the GO and BMP2-GO adsorption were investigated by FTIR spectroscopy. A peak value of 1800/cm confirmed that carboxyl (C = O) played a key role in the BPM2-GO.

### Cellular co-cultures with GO

As shown in Fig. 1d, immunofluorescence was used to show the chondrocytes coexisting with GO particle. A clear difference appeared after 10 days. More importantly, when the GO was co-cultured with the cells, the number of cartilage-cell was increased significantly compared with 1 day around the GO particle.

In this approach, GO is regarded as a confirmed-positive biomedical method for matrix regeneration, as it is known to be able to induce the deposition of cartilage tissue. Moreover, 3D-printed GO scaffolds have been considered as a platform to rebuild a tissue matrix and to be involved in microenvironmental ecology stability. However, the 3D-printed GO scaffold does not simply support biological macromolecules in the matrix. The 3D-printed scaffold also generates intracellular signaling pathways, e.g., bone morphogenetic protein families and transforming growth-factor families [19].

### Morphology of a 3D-printed GO scaffold

**Fig. 2:**
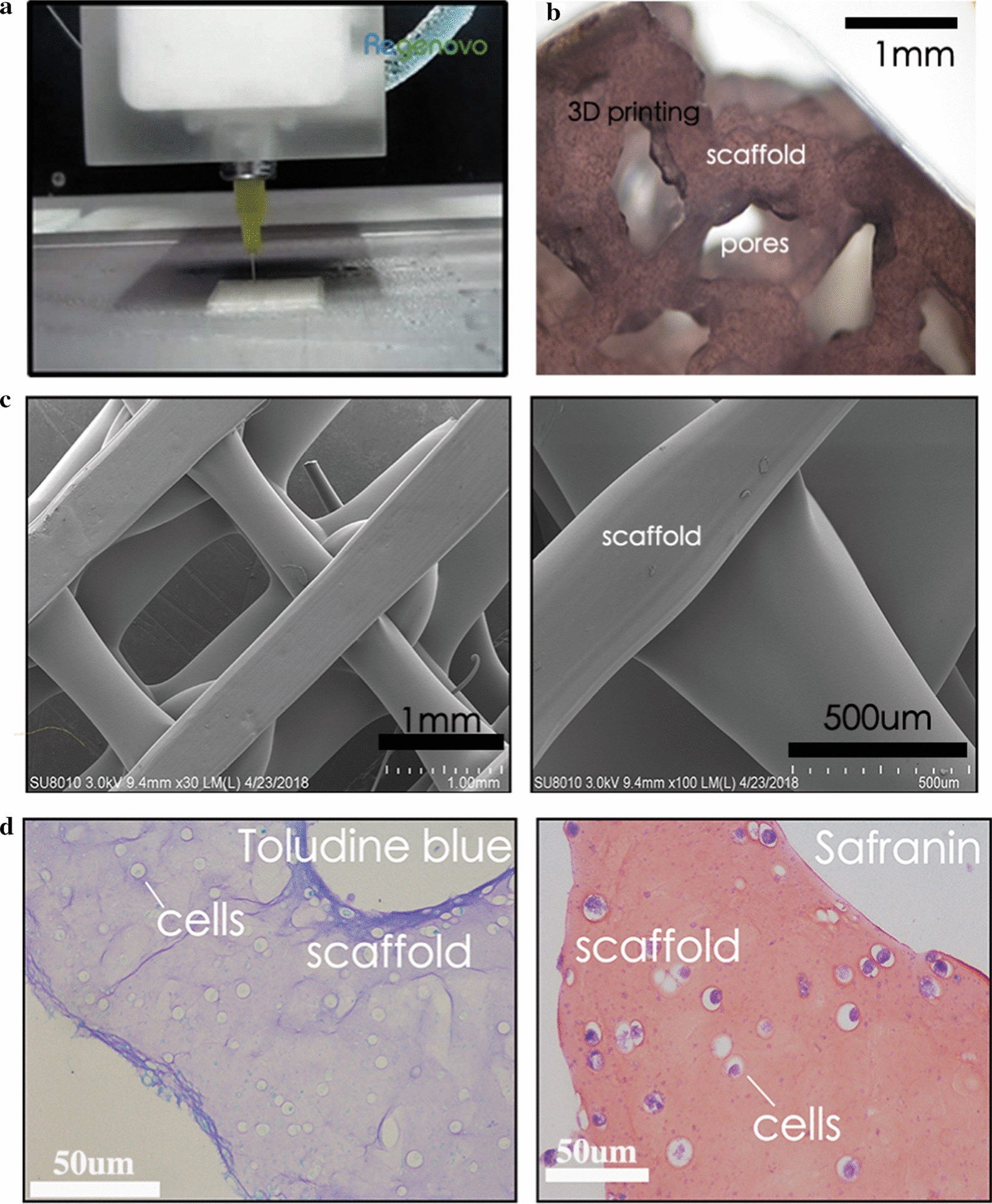
**a** Square design adopted as a simple model of the GO scaffold. **b** Grid pattern of the micro-GO scaffold: Pores existed in the GO scaffold. **c** Printed tissues as viewed using SEM. **d** Toluidine blue and safranin staining of micro-GO scaffold with cartilage cells

A square design was adopted as a simple model of the GO scaffold. Pores appeared and cells were set in the model scaffold. The Regenovo printing platform was adopted in this research. The size of morphological observations were 1 mm (Fig. 2a). Figure 2b, c show the printed tissues, as viewed by the naked eye and under SEM (bar = 100 μm), respectively. It is clear that the 3D-printed GO scaffold displayed a 3D network with high porosity; moreover, the GO was not observed on the surface of the microfibers. Figure 2d shows the toluidine blue and safranin staining of a 3D-printed GO scaffold with cartilage cells (bar = 200 μm).

### 3D printed tissue in vitro

**Fig. 3:**
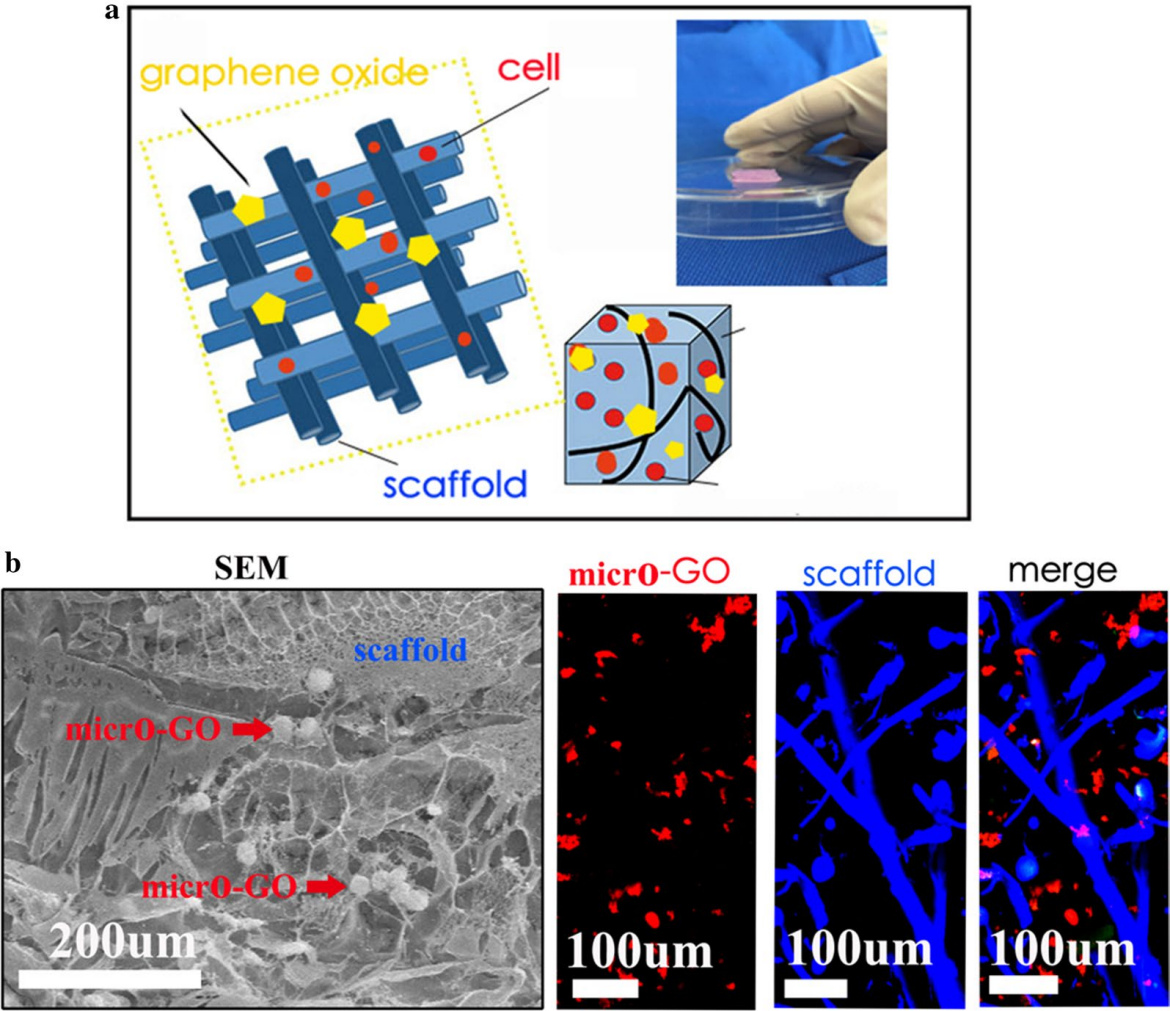
**a** Synthesis of micro-GO with 3d printing scaffold. **b** SEM and Immunofluorescence in vitro evaluation according to the synthesis of micro-GO in scaffold. The scaffold is interweaved into nets and the micro-GO was inserted it

**Fig. 4:**
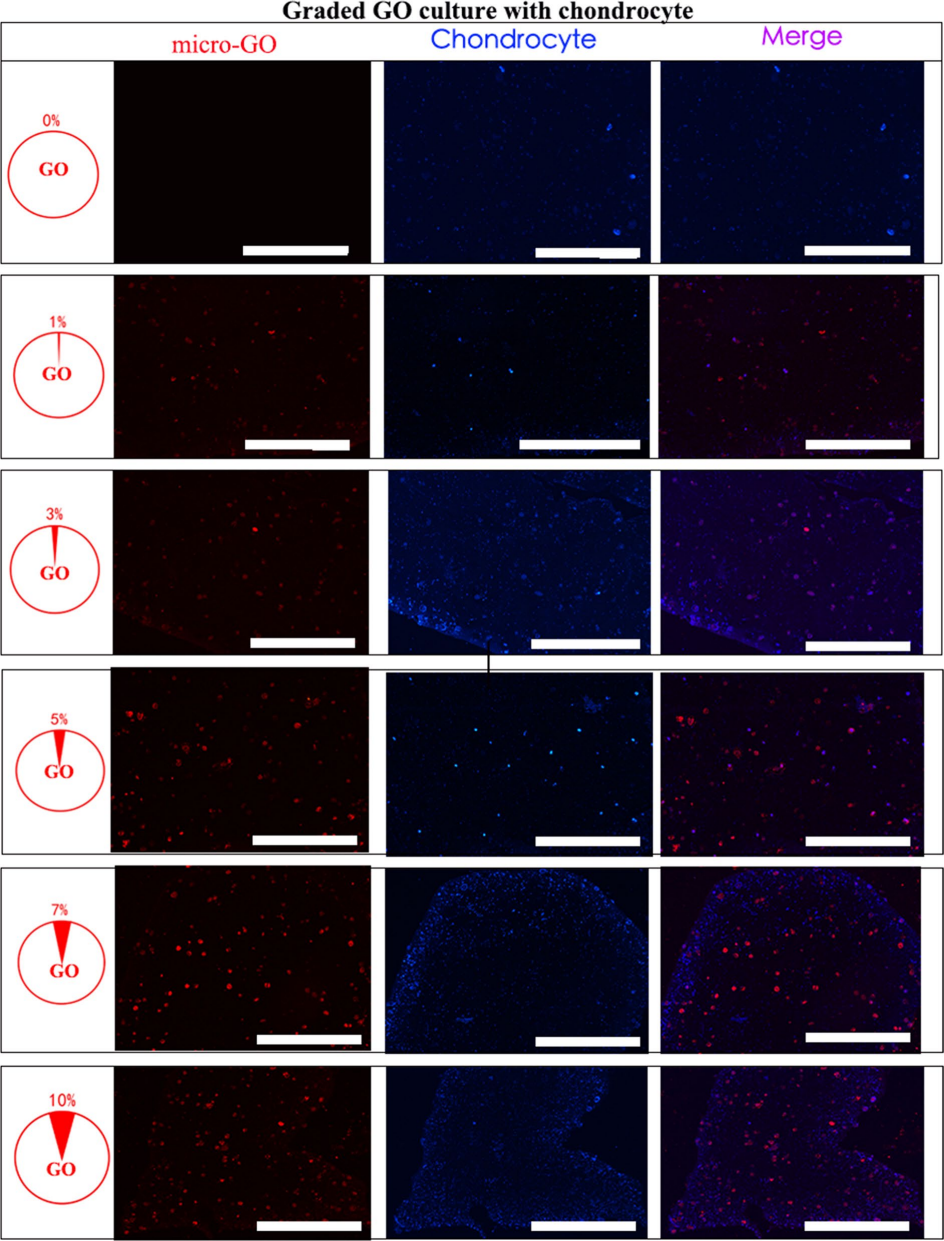
Chondrocyte proliferation revealed by qualitative analysis for chondrocyte cultured on the 3D printing scaffold with graded GO, by 1%, 3%, 5%, and 7% micro-GO scaffold, that for 10% was higher than for the other groups (bar = 200 μm)

**Fig. 5:**
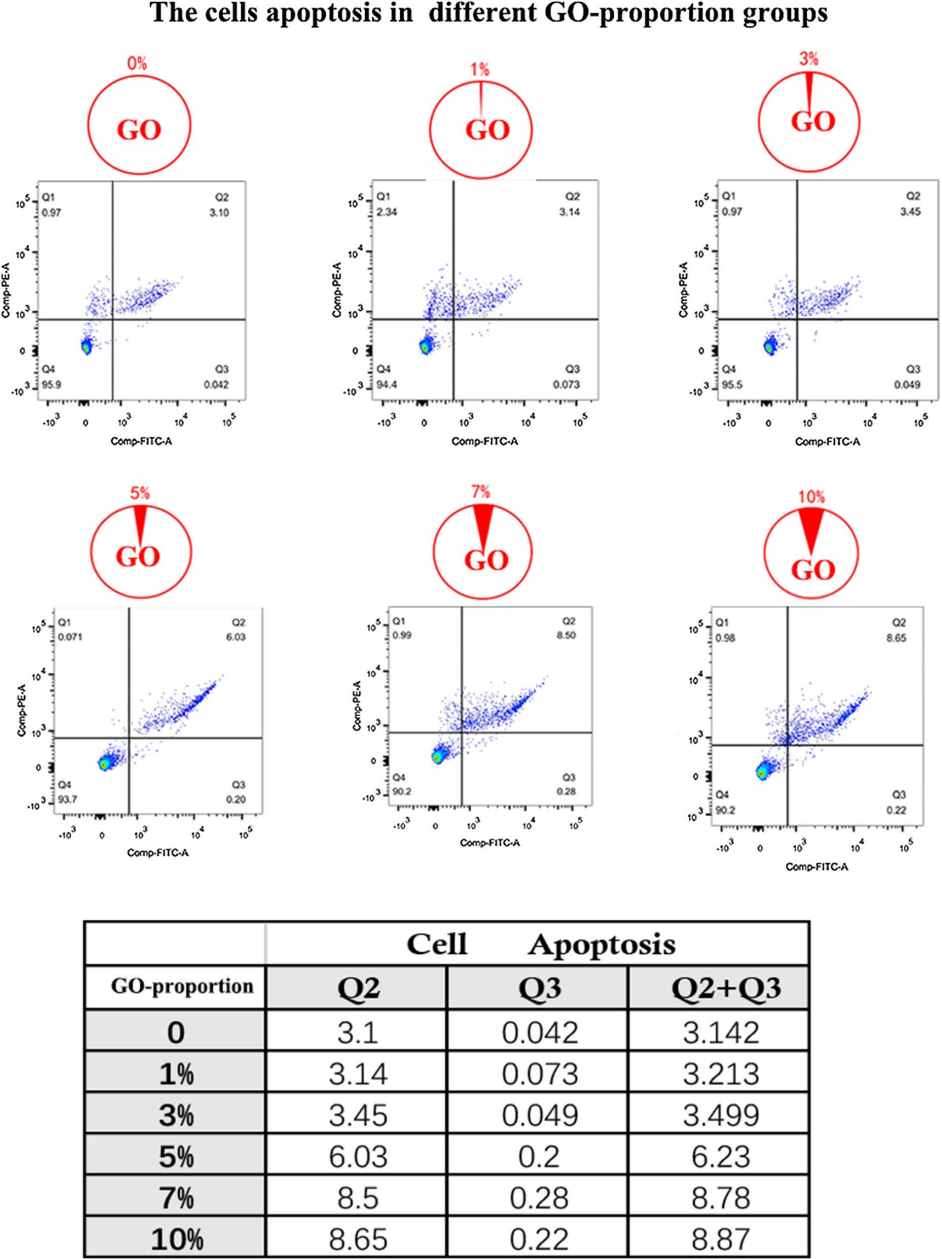
The cells apoptosis in different GO-proportion groups was tested by flow cytometry. Increase of cell apoptosis from 1 to 10% revealed by quantitative analysis from flow cytometry cell apoptosis for chondrocyte cultured

**Fig. 6:**
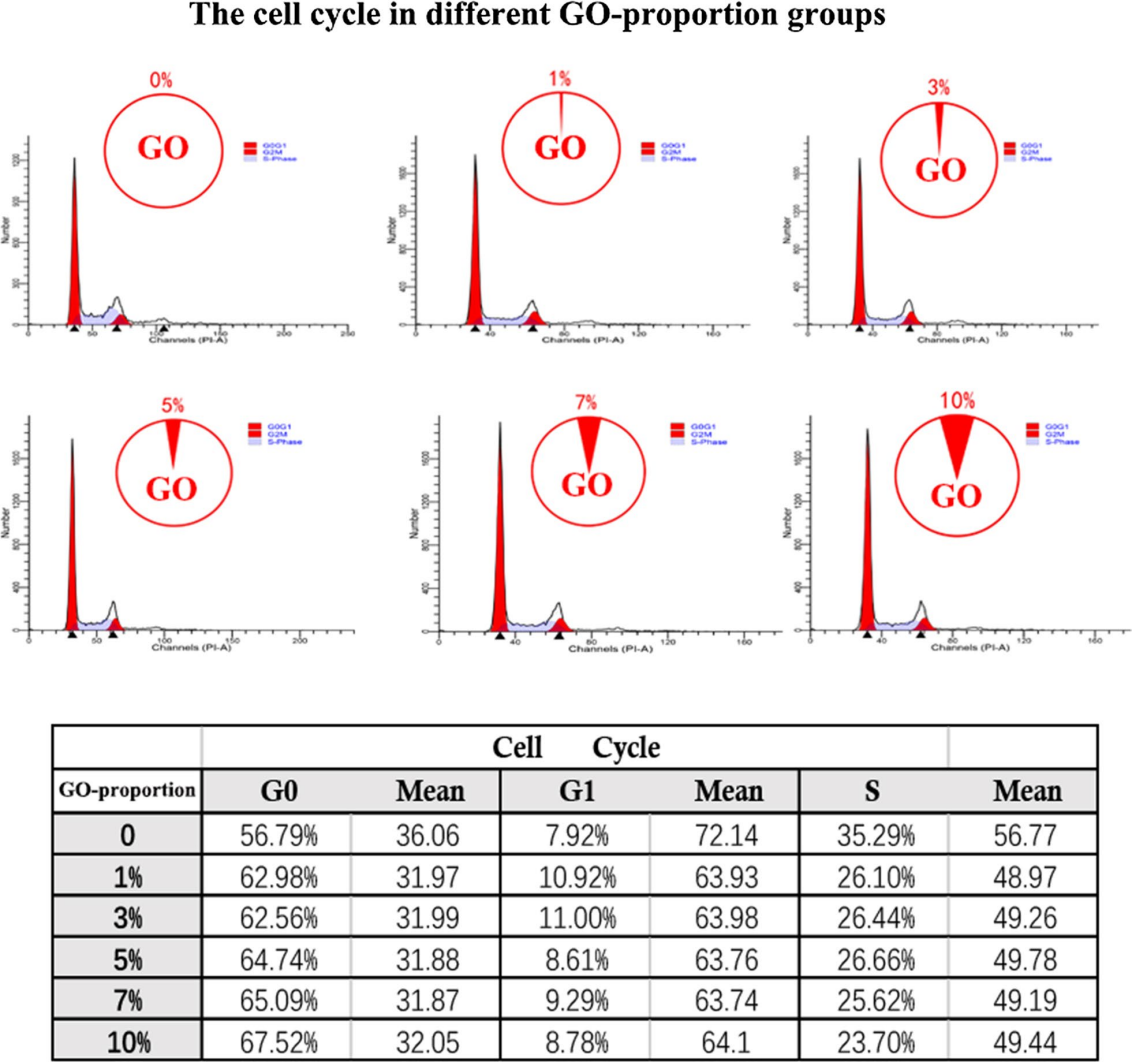
The cell cycle in different GO-proportion groups was tested by flow cytometry in different treatment groups. Decrease of S stage cells from 1 to 10% by quantitative analysis from flow cytometry cell cycle for chondrocyte cultured. Increase of G0 stage cells from 1 to 10% by quantitative analysis from flow cytometry cell cycle for chondrocyte cultured

Figure 3a shows the synthesis of the 3D-printed scaffold with GO. The three dimensional relationship between were them evaluated by SEM and immunofluorescence in vitro. Furthermore, we noticed that the micro GO within the scaffold which is interweaved into nets and the micro-GO was inserted it (Fig. 3b). In a qualitative analysis, 1%, 3%, 7%, and 10% GO was printed in the 3D scaffold respectively. The results suggested that 10% GO exhibited higher chondrocyte proliferation potential than the other groups (Fig. 4). Additionally, the quantitative analysis from flow cytometry cell apoptosis for chondrocyte cultured revealed that the percent of cell apoptosis increased from 1 to 10% (Fig. 5), and the percent of S stage decreased from 1 to 10% (Fig. 6). These results indicate that in the chondrocyte cultured on the 3D-printed scaffold, 10% GO could be better used for collagen synthesis. Meanwhile, cell apoptosis was activated. So, the optimum concentration of GO should correspond with the collagen synthesis to cell apoptosis. However, the optimum concentration of GO requires further research and discussion.

3D printing with GO has offered a broader range of regeneration tissue or tissue matrix production, which can determine the configuration between the seed cells and the matrix [20]. It also supports a solution for tissue-matrix reconstruction. After observing the GO in a tissue microenvironment subsidiary to those extracellular scaffold elements, it is clear that the cell adhesion to 3D tissues in a microenvironment with GO can promote degeneration and regeneration.

In this project, the bionic microenvironment of the cartilage matrix was innovatively reconstructed to further elucidate the potential mechanism. Certain studies from clinics and laboratories have clarified the crucial techniques and fundamental methods of 3D printing with GO tissue procedures and have employed printing instruments for artificial cartilage-tissue simulation models [21]. Furthermore, 3D printing has initiated innovative research on bionic cartilage, including extracorporeal cell and tissue matrix repairs.

### 3D printed tissue in vivo

**Fig. 7:**
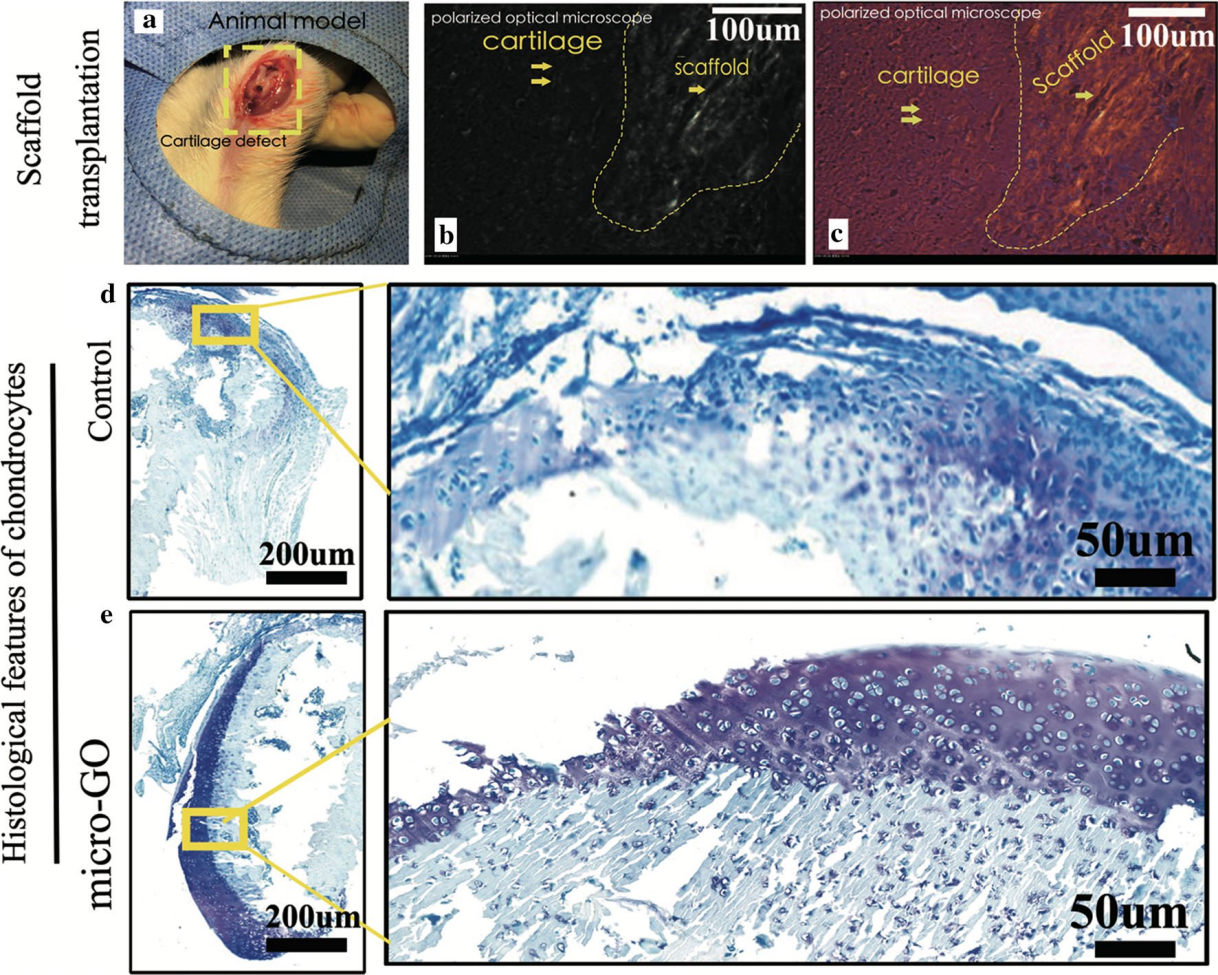
**a** 3D-printed tissue-transplantation surgery on both knees of animal model. **b**, **c**. Polarized optical microscopy result showing the different scaffold zones with cartilage zones. **d**, **e** The histological features of chondrocytes with 3D-bioprinted scaffold transplantations after 6 weeks were presented (bar = 200 μm, 50 μm, respectively)

Polarized optical microscopy was used to show the different cartilage zones in the scaffold (Figs. 7b, c). The histological features of the chondrocytes with 3D-bioprinted scaffold transplantations after 6 weeks are presented in Figs. 7d, e. The cells showed a significant increase in the scaffold, compared with a 3D-printed scaffold with cartilage cells. Furthermore, the chondrocytes were stained with toluidine blue after treatment with the 3D-printed GO scaffold. The new cartilage tissues were mixed with the 3D-printed scaffold.

Our study has clarified that 3D printing with GO is a type of potential bionic platform to satisfy issues after cell installations. As the 3D reconstruction of a cartilage-matrix tissue remains a key issue in bionic cartilage engineering, a stereoscopic scaffold with GO enables the bionic cartilage tissue to be more productive. The 3D-printed platform provided an advanced instrument for exploring intracellular and extracellular cell metabolisms. Moreover, our in vivo research has revealed that 3D printing with GO represents an upcoming and valuable platform for the study of bionic cartilage regeneration, depending on bioengineering and biomanufacturing technologies [20].

### Immunohistochemical staining

**Fig. 8:**
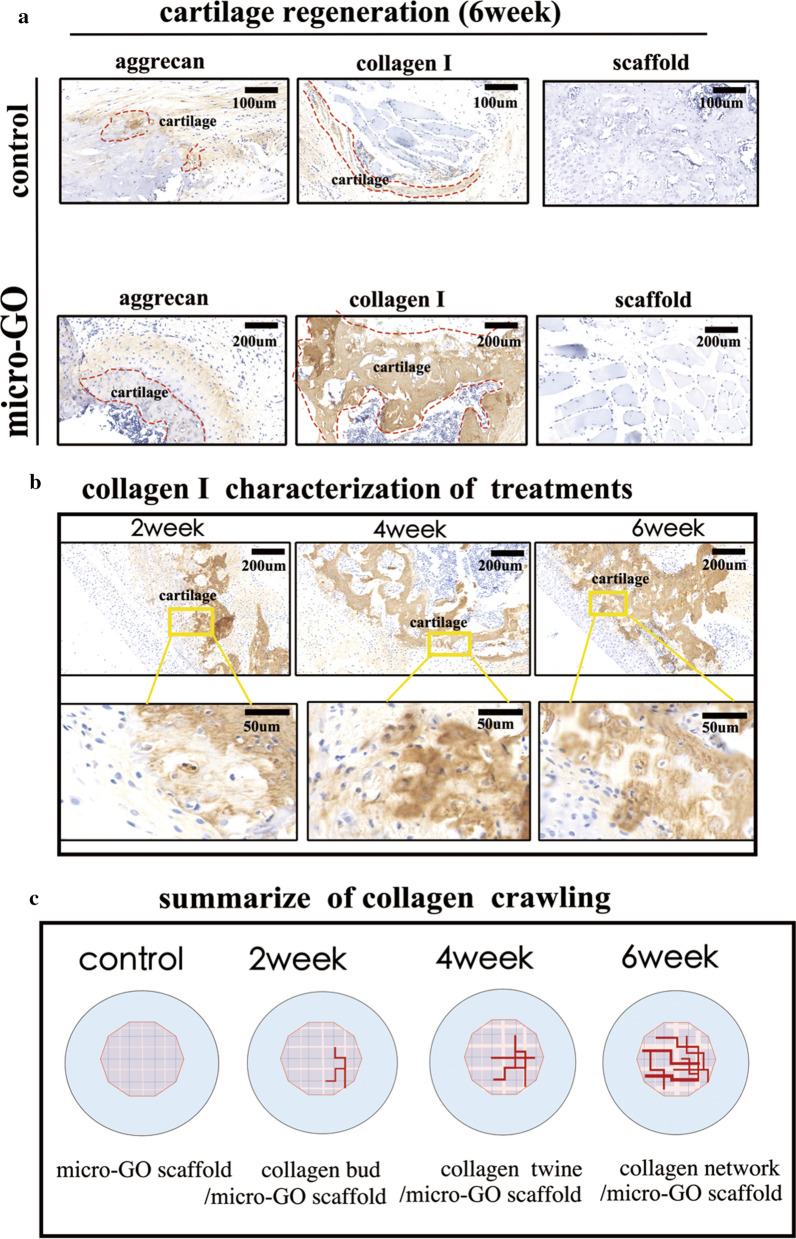
**a** Analysis of immunofluorescence staining with aggrecan and collagen I showing that the cartilage with 3D-printed scaffold only was thinner than that with micro-GO scaffold after 6 week (bar = 200 μm). **b** Significant differences of immunohistochemical collagen I in treatment with micro-GO scaffold from weeks 2 to 6 (bar = 200 μm, 100 μm, respectively). **c** Process of renewed collagen I summarized as follows by the above results: the collagen I bud appeared in week 2, collagen I was twined in week 4, and was net together in week 6. Meanwhile, the scaffold in the matrix collapsed step by step

After the transplantations of the 3D-printed tissue, the stained cartilages can be seen in Fig. 8. In addition, after immunofluorescent staining with aggrecan and collagen I, an analysis showed that the cartilage with a scaffold following the 3D-printed scaffold was thinner than that of the 3D-printed GO scaffold(Fig. 8a). Furthermore, the differences in the immunohistochemical collagen I in the treatment with the 3D-printed scaffold were significant from weeks 2 to 6. The renewed tissue was clustered in the morphology (Fig. 8b).

The cartilage tissue was simulated in the study with the 3D-printed method. The biocompatibility of the scaffold constructed by 3D printing was remarkably significant. Furthermore, the cartilage-regeneration process was simulated and speculated. The newborn cartilage matrix in the matrix was observed to extend along the border of the cartilage and scaffold, and it matured in sequence. The collagen in the matrix expanded and interlaced. After implantation, the scaffold in the matrix collapsed, step by step. Therefore, the 3D-printed scaffold proves a novel platform for the purposeful investigation of the cartilage-matrix regeneration mechanism. This phenomenon has not been reported previously.

3D-printed scaffolds are special 3D materials, in contrast to the regular structure attributed to the protogenesis matrix. The physical structure of the cell attachments with a 3D-printed scaffold is the major cause of matrix deposition. Furthermore, owing to its transparent surface, a cartilage matrix has a high capability for crosslinking with the collagen regeneration. A 3D-printed scaffold can also control the tissue mineralization in a region-specific manner with vascularization in a versatile and scalable approach [22, 23]. Additionally, the adoption can improve the mechanical properties of the scaffold and may act as a structure for maintaining cell growth [24].

Decellularized extracellular matrices, a type of bio-ink resource for cell-laden constructs, can provide an ideal bionic microenvironment that is considered when it comes to the growth of 3D-structured tissue [25]. The advantage of utilizing GO-based material lies in its ability to be processed for tissue-specific bionic applications [26]. For example, electrospun and microsphere sintered scaffolds are more applicable to bone regeneration. Analogously, GO is suitable for ligament-tissue regeneration. Recent investigations have only focused on explaining the potential mechanisms between GO and matrices.

## Conclusion

In conclusion, our data elucidated that 3D-printed scaffolds are powerful platforms for further investigations on the regeneration mechanism of cartilage matrices. 3D printing with GO is particularly proposed for cartilage-matrix regeneration. It has expanded our knowledge of how a newborn cartilage matrix extends and matures in sequence, which is accompanied by the step-by-step collapse of the scaffold.

However, because of the multiformity and biological interaction of the polymers, GO has not yet been fully investigated. The details of its biological signal responses at the matrix boundary have been insufficient and require further investigations. Therefore, 3D printing with GO as a smart macromolecular material in biomedical applications should be established in a subsequent study.

## Data Availability

The data and materials used in the study are available from the corresponding author.
